# Cu and Boron Doped Carbon Nitride for Highly Selective Oxidation of Toluene to Benzaldehyde

**DOI:** 10.3390/molecules200712686

**Published:** 2015-07-13

**Authors:** Hongling Han, Guodong Ding, Tianbin Wu, Dexin Yang, Tao Jiang, Buxing Han

**Affiliations:** Beijing National Laboratory for Molecular Sciences (BNLMS), Institute of Chemistry, Chinese Academy of Sciences, Beijing 100190, China; E-Mails: hanhll@iccas.ac.cn (H.H.); dinggd@iccas.ac.cn (G.D.); wtb@iccas.ac.cn (T.W.); yangdx@iccas.ac.cn (D.Y.)

**Keywords:** toluene, selective oxidation, graphitic carbon nitride, benzaldehyde

## Abstract

A novel Cu and boron doped graphitic carbon nitride catalyst (Cu-CNB) was synthesized using cheap precursors and systematically characterized. The selective oxidation of toluene proceeded very smoothly over the catalyst at 70 °C using tert-butyl hydroperoxide (TBHP) as the oxidant to exclusively afford benzaldehyde. The catalyst can be used for at least five cycles without decrease in activity and selectivity.

## 1. Introduction

Selective oxidation of primary C-H bonds to produce useful functional chemicals is of great importance in chemical transformations [1,2,3,4,5,6,7]. As a typical alkyl aromatic molecule, toluene can be oxidized to benzyl alcohol, benzaldehyde, benzoic acid and benzyl benzoate, which are all commercially important intermediates in the production of fine chemicals such as pharmaceuticals, foodstuff, dyes, perfume and resins. Commercial benzaldehyde is mainly produced by the chlorination of toluene followed by saponification which suffers from several drawbacks such as high energy consumption, low yields, and the danger associated with the use of Cl_2_ [8]. Consequently, there is significant interest in the design of green and efficient heterogeneous catalysts for the selective oxidation of toluene. Recently, several catalyst systems including Au-Pd nanoparticles [9,10], HDPA-Fe_3_O_4_ [11], CuCr_2_O_4_ spinel [12], and Cu-Mn oxides [13] have been developed for the selective oxidation of toluene. Generally, the catalytic systems suffered from various drawbacks such as, harsh conditions, low conversion, and low selectivity especially to benzaldehyde. Selective catalytic oxidation of toluene to benzaldehyde still remains a great challenge because of the over oxidation of the as-formed benzaldehyde with increased reactivity relative to toluene [14,15].

Graphitic carbon nitride (g-C_3_N_4_), due to its unique structure and thus excellent properties, such as semiconductivity and nitrogen richness, can often be used as a metal-free catalyst or catalyst support for heterogeneous catalytic oxidation [16,17,18,19,20,21,22,23]. However, it was shown that the more ideal bulk carbon nitride solids had poor catalytic performance in some catalytic processes, while more disordered polymeric versions showed better activity, as structural defects or surface terminations seemed to play a key role for the catalytic activation [24]. To enhance the performance of carbon nitride both as a support and as a catalyst, the specific surface have to be enhanced. Wang *et al.* have shown that CNB exhibited good catalytic selectivity for the oxidation of toluene to benzaldehyde using H_2_O_2_ as oxidant [25]. Li *et al*. reported the selective oxidation of toluene to benzaldehyde over mesoporous g-C_3_N_4_ using O_2_ under solvent-free conditions, giving high selectivity but relatively low conversion [26]. Our group have demonstrated a series of metal doped graphitic carbon nitride (Cu-, Fe-, V-, Co-, and Ni-g-C_3_N_4_), among which V-g-C_3_N_4_ was found to be the most efficient catalyst for the direct hydroxylation of benzene to phenol with 100% selectivity using H_2_O_2_ as the oxidant [27]. However, among the few reports about the selective oxidation of toluene, these reactions often needed harsh conditions or suffered from low conversions. Catalysis using copper oxides is well established in the literature for liquid-phase oxidations of hydrocarbons and alcohols [28,29,30,31,32] because Cu possesses an interesting redox cycle (Cu^2+^/Cu^+^) that is amenable to facilitating free-radical oxidation reactions.

In this work, a novel Cu and B doped graphitic carbon nitride catalyst was synthesized and applied for the selective oxidation of toluene. The simple, commercially available room-temperature ionic liquid (IL), 1-cyanopropyl-3-methylimidazolium tetrafluoroborate (CpmimBF_4_) was used as an additive, urea and Cu(NO_3_)_2_•3H_2_O as the precursors for the synthesis of Cu-CNB catalyst. The structure of the Cu-CNB catalyst was characterized systematically using N_2_ adsorption-desorption, Fourier transform infrared spectroscopy (FT-IR), powder X-ray diffraction (XRD), and X-ray photoelectron spectroscopy (XPS), inductively coupled plasma-atomic emission spectrometry (ICP-AES) techniques. The catalyst showed stable and superior performance in the oxidation of toluene with good conversion and benzaldehyde as the sole product.

## 2. Results and Discussion

### 2.1. Catalytic Performance of the Cu-CNB Catalyst for the Selective Oxidation of Toluene to Benzaldehyde

The novel Cu and boron doped graphitic carbon nitride catalyst has been successfully synthesized by using urea, boron-containing IL and Cu(NO_3_)_2_•3H_2_O as the precursor through a facile and efficient method, denoted as Cu-CNB. The selective oxidation of toluene on the Cu-CNB catalyst by TBHP or H_2_O_2_ was tested in a Teflon lined autoclave with acetonitrile as solvent. The desired amount of catalyst, toluene and oxidant were dispersed in acetonitrile, followed by the temperature of the autoclave ramped to reaction temperature and then the oxidation reaction started. The products were taken out from the reactor after desired reaction time and analyzed by an Agilent 6820 equipped with a flame ionization detector (FID) using anisole as the internal standard.

The performance of the Cu-CNB catalyst for the oxidation of toluene to benzaldehyde was studied using *tert*-butyl hydroperoxide (TBHP) as the oxidant, and the results are given in Table 1. Good conversion of toluene and excellent benzaldehyde selectivity were obtained. When the reaction was conducted in the presence of TBHP but without the catalyst, no detectable conversion of toluene was found (Entry 1). When the oxidation of toluene was conducted in the presence of the neat support, no reaction was observed under our reaction conditions (Entries 2, 3), which proved that Cu species were necessary for the reaction. To discuss the role of the boron in the catalyst, we also synthesized the Cu-C and Cu-C_3_N_4_ for the selective oxidation of toluene. It can be seen from Table 1 (Entries 4, 5), that both the Cu-C and Cu-C_3_N_4_ can selectively catalyze toluene oxidation to benzaldehyde, but the conversions were lower than that of Cu-CNB. It has been reported by Wang [25] that the boron functional groups on the surface might act as strong Lewis acid sites, which would complement the basic nitrogen sites into a bifunctional catalysis. On the other hand, Kiwi-Minsker and coworkers [33] reported that basic sites would increase the selectivity of benzaldehyde but decrease the activity of the catalyst. Thus it can be speculated that boron on the surface of the Cu-CNB catalyst might have acted as Lewis acid sites, complementing the basic nitrogen sites, and thus increased the catalytic activity of the catalyst. It is worth noting that the Cu-CNB material could catalyze the oxidation of toluene to benzaldehyde with >99% selectivity (Entries 6–9). Under optimized reaction conditions, Cu-CNB showed 6.3% conversion of toluene (Entry 9) with >99% selectivity to benzaldehyde.

**Table 1:** Selective oxidation of toluene on Cu and boron doped graphitic carbon nitride catalyst (Cu-CNB) with tert-butyl hydroperoxide (TBHP) as oxidant ^a^.

Entry	Catalysts	*n*_toluene_/*n*_TBHP_	Temperature/°C	C/%	S/%
1	--	1:2.5	70	0	0
2	C_3_N_4_	1:2.5	70	0	0
3	CNB	1:2.5	70	0	0
4	Cu-C	1:2.5	70	1.42	>99
5	Cu-C_3_N_4_	1:2.5	70	1.59	>99
6	Cu-CNB	1:2.5	70	4.3	>99
7	Cu-CNB	1:2.5	80	4.8	>99
8	Cu-CNB	1:4	70	5.5	>99
9 ^b^	Cu-CNB	1:4	70	6.3	>99

^a^ C = toluene conversion, S = benzaldehyde selectivity. Typical reaction conditions are as follow unless stated: 30 mg of catalyst, 10 mmol of toluene, 25 mmol of TBHP, 3 mL of acetonitrile, 70 °C, 10 h. ^b^ Reaction time, 16 h.

**Figure 1:**
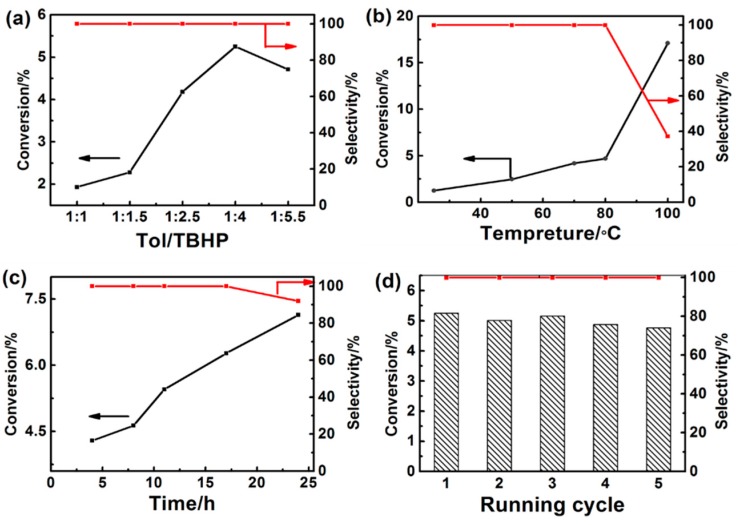
(**a**) The influence of the amount of TBHP on the selective oxidation of toluene over Cu-CNB (70 °C, 10 h); (**b**) The influence of reaction temperature on the selective oxidation of toluene over Cu-CNB (25 mmol of TBHP, 10 h); (**c**) The influence of reaction time on the selective oxidation of toluene over Cu-CNB (40 mmol of TBHP, 70 °C); (**d**) Reuse of the catalyst Cu-CNB for the selective oxidation of toluene (40 mmol of TBHP, 70 °C, 10 h). Reaction conditions: 30 mg of catalyst, 10 mmol of toluene, 3 mL of acetonitrile.

The influence of the amount of TBHP on the selective oxidation of toluene over the Cu-CNB catalyst was investigated (Figure 1a). The conversion of toluene first increased as the molar ratio of TBHP to toluene increased to 4 and then decreased, with the selectivity of benzaldehyde remained larger than 99% in the whole range of TBHP amount tested. We also studied the influence of reaction temperature on the conversion and selectivity of the oxidation reaction over the Cu-CNB catalyst, as shown in Figure 1b. As expected, the conversion increased with reaction temperature. The selectivity of benzaldehyde was larger than 99% before 80 °C. However, when the reaction temperature was higher than 80 °C, the selectivity of benzaldehyde sharply decreased and CO_x_ was detected, indicating that over oxidation of toluene occurred at the higher temperature. The influence of time on the reaction was shown in Figure 1c. Increasing reaction time enhanced the conversion of toluene, and the conversion reached 6.3% at 16 h with the selectivity of benzaldehyde >99%. When the reaction time was further prolonged to 24 h, the conversion of toluene was increased to 7.1%, but the selectivity declined to 92%, which could be attributed to the over oxidation of benzaldehyde. The reusability of the catalyst for the reaction was evaluated. The results showed that the selectivity to benzaldehyde was not changed and the conversion of the toluene did not decrease considerably over five catalytic cycles (Figure 1d).

There are three main pathways for the oxidation of toluene in terms of the oxidants involving molecular oxygen, hydrogen peroxide, and TBHP. Hutchings and co-workers have shown that Au-Pd alloy nanoparticles were very effective for the selective oxidation of toluene with molecular oxygen, giving high selectivity to benzyl benzoate [9]. However, when using TBHP as the oxidant, the above Au-Pd alloy catalysts performed with 4.4% conversion of toluene and yielded a mixture of benzyl alcohol, benzaldehyde and benzoic acid without obvious product selectivity [10]. A similar phenomenon was observed over Cu-CNB catalyst in that different oxidants led to different products. When H_2_O_2_ was used as the oxidant, several products, benzyl alcohol, benzaldehyde, benzoic acid, *o*-cresol, *p*-cresol, and methyl-*p*-benzoquinone were detected (Table 2). Interestingly, as shown in Table 1, benzaldehyde was the only product in the reaction when TBHP was used as the oxidant. Different free radicals and microenvironments produced when the TBHP or H_2_O_2_ contacted the catalyst would cause the totally different products.

**Table 2:** Selective oxidation of toluene on Cu-CNB with H_2_O_2_ as oxidant ^a^.

Entry	Catalyst/mg	Time/h	T/°C	Selectivity/%					
				BOL	BAL	BAC	*o*-cresol	*p*-cresol	MPB
1	20	20	70	8.5	37	31.2	10.9	7.1	5.3
2	30	20	70	17.7	61.7	0	10.5	6.3	3.9
3	50	20	70	10.8	38.7	22.8	11.9	8.1	7.7
4	90	20	70	6.7	36.1	19.5	15.6	13.3	8.9
5	30	8	70	18	52.1	5.7	9.3	7.3	7.6
6	30	16	70	17.1	61.5	0	8.1	6.3	7.1
7	30	24	70	17.3	63.7	0	8.5	6.8	3.7
8 ^b^	30	24	70	23.4	37.7	28	2.8	0	8.1
9	30	4	40	23.1	18.8	0	34	24.1	0
10	30	4	60	7.6	36.4	0	26.6	20.7	8.7
11	30	4	70	14.9	46.3	14.2	9.4	7.6	7.7

^a^ Selectivity based on the liquid products detected, BOL = benzyl alcohol, BAL = benzaldehyde, BAC = benzoic acid, MPB = Methyl-*p*-benzoquinone. Typical reaction conditions were as follows until otherwise stated: 10 mmol of toluene, 29.4 mmol of H_2_O_2_, 3 mL of acetonitrile; ^b^ 49 mmol of H_2_O_2_.

**Scheme 1:**
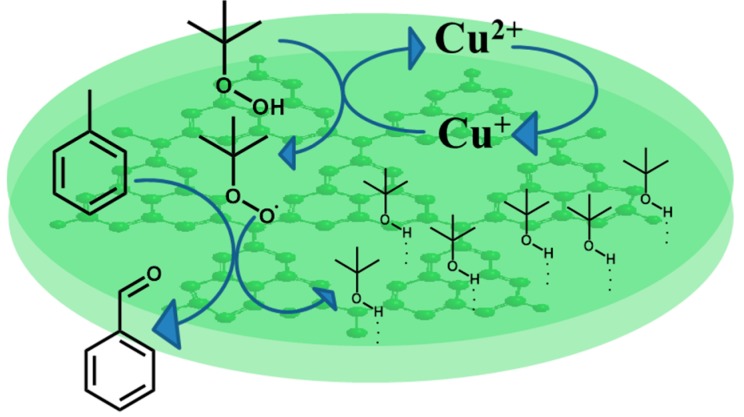
Representative scheme of the oxidation of toluene on the catalyst Cu-CNB surface.

It is known that there are abundant nitrogen content and uncondensed -NH_2_ and -NH- groups on the surface of g-C_3_N_4_ [34]. Tert-butyl alcohol formed during the oxidation process can be easily absorbed on the catalyst surface through hydrogen bonding, which endowed the catalyst with hydrophobic surface, and thus toluene can access the Cu-CNB. Moreover, the tert-butyl alcohol absorbed on the surface of the catalyst might restrict the possible interaction between -NH_2_ or -NH- and the produced benzaldehyde, resulting in the relatively polar molecule benzaldehyde could easily escape from the catalyst. It can be easily understood that if catalyst surface absorbed too much tert-butyl alcohol molecules, the big steric hindrance of tert-butyl alcohol would restrict the diffusion of toluene to catalyst surface, thus reduces the toluene conversion (Scheme 1).

### 2.2. Structure Characterization of the Cu-CNB Catalyst

**Figure 2:**
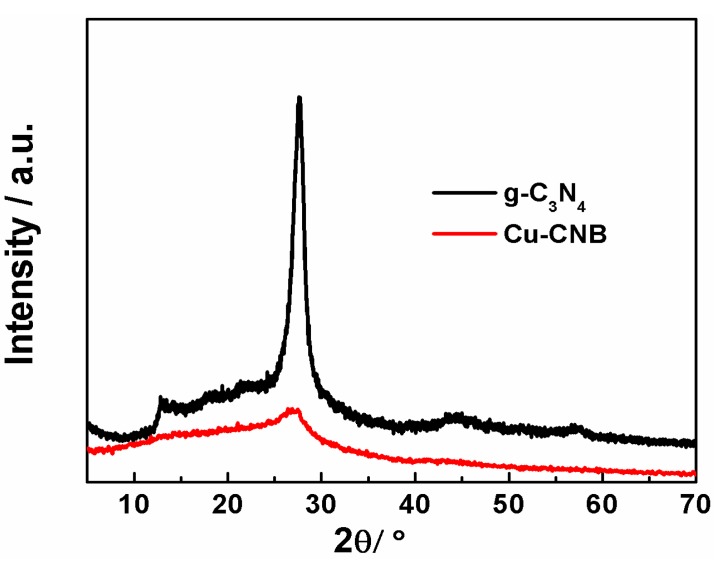
The XRD patterns of the Cu-CNB.

The structure of the Cu-CNB catalyst was characterized systematically using N_2_ adsorption-desorption, FT-IR, XRD, XPS, and ICP-AES techniques. XRD patterns of g-C_3_N_4_ and the Cu-CNB catalyst were shown in Figure 2. It could be seen that the diffraction peak at around 27.4°, which represented the typical (002) interlayer-stacking peak of graphite-like structure [35], was not as strong as the pure g-C_3_N_4_, as well as the peak at 12.9° corresponding to in-plane ordering of *tri*-s-triazine units. Thus, it can be speculated that incorporating Cu and B into C_3_N_4_ disturbed the ordered structure of the material, and hence reduced the crystallinity. In addition, there was no diffraction peak attributed to the crystalline copper species such as cupric oxides, cupric nitrides, or cupric carbides observed in Figure 2, indicating that the copper species stayed chemically coordinated to the C_3_N_4_ matrix, most likely in the form of copper-N bonds, as reported in previous work [36].

**Figure 3:**
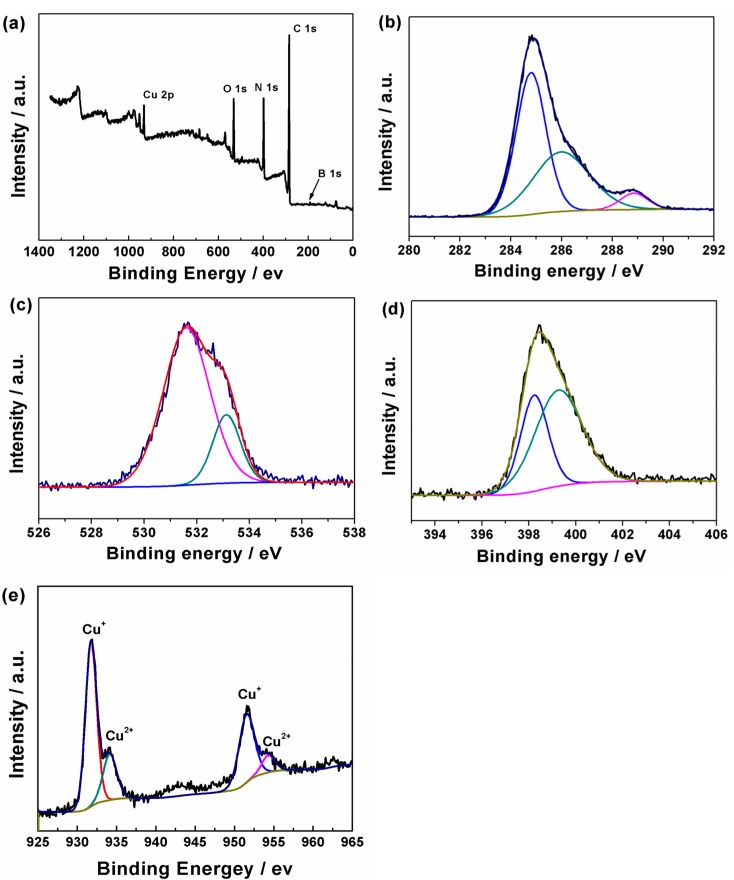
XPS spectra of Cu-CNB: (**a**) Cu-CNB; (**b**) C 1s; (**c**) O 1s; (**d**) N 1s; and (**e**) Cu 2p.

It could be seen that the main elements on the surface of the catalyst were C, N, O, B and Cu (Figure 3a). The B1s binding energy peak of the catalyst was centered at 191.3 eV, as reported in the literature [25], indicating the formation of B-N. The C 1s profile was separated into three peaks 284.8, 286.0 and 288.9 eV, as shown in Figure 3b, indicating the existence of C-N, C-O and C=O on the catalyst surface [37]. Figure 3c exhibited the O 1s profile separated into three peaks, and the peaks at 531.6 and 533.1 eV represented C=O and C-O, respectively. The O species might be involved in during the calcination and store processes. The N1s XPS spectrum (Figure 3d) included two peaks at 399.3 and 398.2 eV, which could be assigned to tertiary nitrogen atoms bonded to carbon atoms in the form of N-(C)_3_ or H-N-(C)_2_ and C=N-C bonding [38], respectively. Both the broad Cu 2p_3/2_ and Cu 2p_1/2_ peaks have been separated into two peaks (see Figure 3e). For the peaks of Cu 2p_3/2_ at 934.2 and 931.8 eV were attributed to the Cu^2+^ and Cu^+^, respectively. In addition, there was no peak in the O 1s XPS spectrum belonged to the Cu-O, thus it can be speculated that the copper atoms were bonded with nitrogen atoms, in accordance with the XRD result.

**Figure 4:**
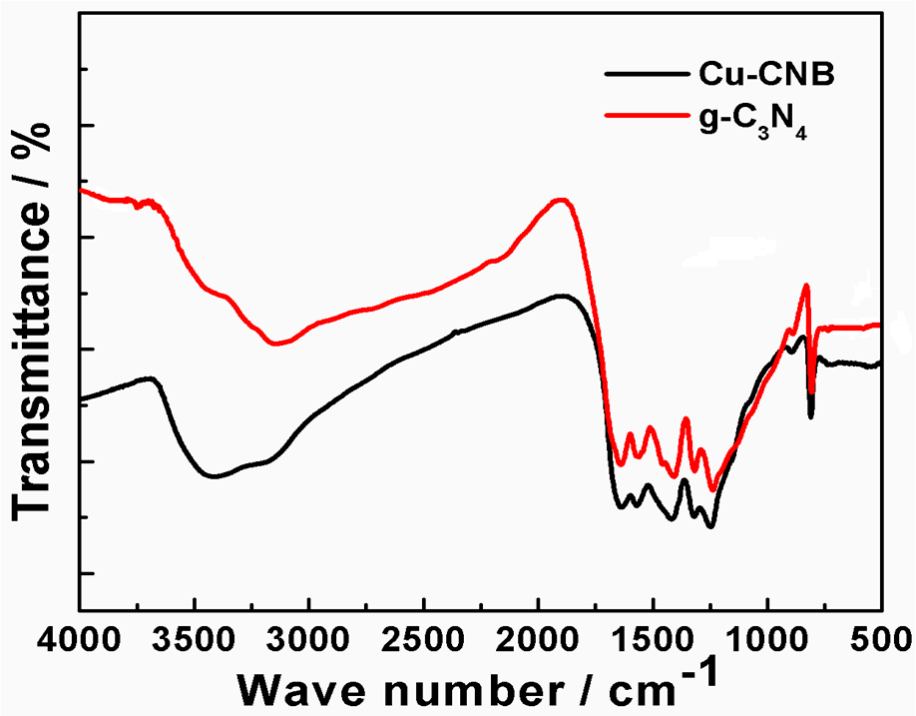
The FT-IR spectra of g-C_3_N_4_ and Cu-CNB.

FT-IR spectra (Figure 4) of the Cu-CNB and g-C_3_N_4_ samples showed similar vibration peaks. The infrared absorption band in the region of 1200–1650 cm^−1^ represented the typical C-N heterocycle stretches, and the peaks at 810 cm^−1^ corresponded to the breathing mode of the tri-s-triazine units, confirming the typical structure of g-C_3_N_4_ retained. In addition, the typical vibration of B-N at 1370 cm^−1^ was presumably overlapped by that of the C-N stretches [39]. The absorption peaks at 3149 cm^−1^ and 3426 cm^−1^ represented the stretching vibration of NH- and OH-, respectively. Furthermore, the vibration of NH_2_- was probably overlapped by that of the OH- stretches.

The nitrogen adsorption-desorption result showed that the Cu-CNB had a BET surface area of 30.1 m^2^/g. It can be speculated that the decrease in BET surface area of Cu-CNB compared with that of g-C_3_N_4_ (96 m^2^/g) [34] was attributed to the insert of Cu and B into the matrix. Elemental analysis revealed that the nitrogen content of the catalyst is approach the ideal C_3_N_4_ (the C/N ratio was around 0.72, and the theoretical C/N ratio of bulk C_3_N_4_ material is 0.75). The Cu content determined by ICP-AES was 1.32 mmol/g (8.39 wt %), which was close to the calculated value based on the feedstock added. The B, O, and H contents were 0.23 wt %, 5.81 wt % and 1.15 wt %, respectively.

## 3. Experimental Section

### 3.1. General Information

Urea (A. R. grade) and toluene (HPLC grade) were purchased from Xilong Chemical Factory (Shantou, China). 1-cyanopropyl-3-methylimidazolium tetrafluoroborate (CpmimBF_4_) were purchased from Centre of Green Chemistry and Catalysis, Lanzhou Institute of Chemical Physics, Chinese Academy of Sciences (purity > 99%, Lanzhou, China). Cu(NO_3_)_2_•3H_2_O (A. R. grade) and acetonitrile (A. R. grade) were obtained from Sinopharm Chemical Reagent Co. Ltd. (Beijing, China). TBHP solution (70 wt %) and benzaldehyde (A. R. grade) were provided by Alfa Aesar Co., Ltd. (Tianjin, China). All of the chemicals were analytical grade and used without further purification.

Sample analysis was operated on an Agilent 6820 gas chromatography equipped with a flame ionization detector (FID) and a HP-5 capillary column (30 m × 0.25 mm × 0.25 μm, Agilent Technologies Singapore (Sales) Pte Ltd., Singapore, Singapore). Identification of the products was carried out on SHIMADZU GCMS-QP2010 Gas Chromatograph-Mass Spectrometer (GC-MS, Shimadzu Co. Ltd., Shanghai, China). FT-IR spectra were recorded on Bruker Tensor 27 spectrometer (Bruker Corporation, Beijing, China) with a resolution of 1 cm^−1^ and 32 scans. XRD were recorded on Rigaku D/max-2500 X-ray diffractometer (Rigaku Corporation, Tokyo, Japan) using CuKa radiation (λ = 0.15406 nm). The tube voltage was 40 kV and current was 200 mA. Pore volumes and Brunauer–Emmett–Teller (BET) surface areas were measured on a Micromeritics ASAP 2020 sorptometer (Micromeritics Instrument Ltd., Shanghai, China) by using nitrogen adsorption at 77 K. XPS were obtained with an ESCALab220i-XL electron spectrometer from VG Scientific using 300 W Al Kα radiation (Thermo Electron Corporation, London, UK). The base pressure was about 3 × 10^−9^ mbar. The binding energies were referenced to the C1s line at 284.8 eV from adventitious carbon. The content of the Cu, C, N and B in the catalyst was analyzed by a PROFILE SPEC ICP-AES (Leeman, Beijing, China).

### 3.2. Preparation of the Catalyst Cu-CNB

The catalyst Cu-CNB was prepared with similar procedures in our previous work [27]. Typically, 12.0 g of urea, 0.1 g of CpmimBF_4_ and 0.25 g of Cu(NO_3_)_2_•3H_2_O were dissolved in 15 mL of distilled water and stirred at 80 °C for 1 h. Then the mixture was heated at 100 °C until the water was completely evaporated. After dried in a vacuum oven at 60 °C for 8 h, the resultant blue solids were then slowly heated at a rate of 3.0 °C/min to reach a temperature of 300 °C, and tempered at this temperature for 2 h in a flowing-nitrogen atmosphere. The mixture was then continuously heated to 550 °C over 1.5 h, and tempered at this temperature for 4 h. This was followed by cooling the sample naturally to room temperature with nitrogen gas. The final powder was collected and labeled as Cu-CNB.

### 3.3. Procedures for the Selective Oxidation of Toluene to Benzaldehyde

The oxidation of toluene over Cu-CNB sample was carried out in a Teflon-lined stainless steel reactor (15 mL total volume) using acetonitrile as the reaction solvent. 1 mL of toluene, 0.03 g of catalyst, desired amounts of 70% *tert-*butyl hydroperoxide in water solution or 30% hydrogen peroxide in water solution and 3 mL of acetonitrile were introduced into the reactor. Then the reactor was heated up to 70 °C with stirring and the oxidation reaction started. The reaction mixture was cooled to room temperature after a desired reaction time. For chemical analysis, a sufficient amount of ethanol was added after reaction which led to homogeneous solution with catalyst particles suspended. Identification of the products and reactant was done using a GC-MS as well as by comparing the retention times to respective standards in GC traces. Quantitative analysis was performed using gas chromatography equipped with a FID detector and a HP-5 capillary column with anisole as the internal standard. In the experiments to test the reusability of the catalyst, the Cu-CNB was separated by centrifugation and the precipitate was washed with ethanol for 3 times. The obtained catalyst was dried in vacuum and refilled for the next run.

## 4. Conclusions

In summary, Cu-CNB can be synthesized through a facile method using cheap raw materials. The catalyst can accelerate the selective oxidation of toluene to benzaldehyde effectively using TBHP as oxidant at 70 °C. The benzaldehyde is the only product as the conversion of toluene is less than 6.3%, and the catalyst can be reused at least 5 times without a considerable decrease in catalytic efficiency. We believe that the easily prepared, highly efficient catalyst has great potential of application, and modification of g-C_3_N_4_ with various heteroatoms is a promising route to design novel catalysts.
